# Robot Technology for Pork and Beef Meat Slaughtering Process: A Review

**DOI:** 10.3390/ani13040651

**Published:** 2023-02-13

**Authors:** Juntae Kim, Yun-Kil Kwon, Hyoun-Wook Kim, Kuk-Hwan Seol, Byoung-Kwan Cho

**Affiliations:** 1 Department of Biosystems Machinery Engineering, Chungnam National University, Daejeon 34134, Republic of Korea; 2 XCore System Co., Ltd., Sejong 30141, Republic of Korea; 3 Animal Products Research and Development Division, National Institute of Animal Science, Wanju 55365, Republic of Korea; 4 Department of Smart Agricultural Systems, Chungnam National University, Daejeon 34134, Republic of Korea

**Keywords:** slaughterhouse automation, robotization, beef, pork, smart slaughterhouse

## Abstract

**Simple Summary:**

This review describes an updated description of robot technology for slaughterhouse automation. The robot technology used in the pork and beef slaughterhouse and details of the visceral laparotomy, carcass preprocessing, and deboning robot technology were introduced. A recent novel slaughter method, meat factory cell (MFC), developed for small-scale slaughterhouse automation, was reviewed, in which more detailed research is required for practical application. New sanitary legislation for the design of robots is required, and standards for system development should be established and updated for slaughterhouse automation. This study is expected to be used for the establishment of the future automation strategy and the introduction of equipment suitable for automated slaughterhouses.

**Abstract:**

Recently, many slaughterhouses have begun to introduce automation and quality evaluation sensing equipment to the slaughter processing line to overcome insufficient human resources, improve the efficiency of the slaughter process, and standardize meat quality. Various processing instruments and sensing technologies may be used depending on the livestock to be slaughtered, but a standardized process design for a smart slaughterhouse remains to be established. Slaughterhouses are becoming more industrialized, leveraging data collection and analysis to drive growth and increase production. Therefore, slaughterhouse automation is essential for meeting production demand, and an optimized design suitable for the size of each company is required to maximize economical equipment and systems. We introduce robot technology used in the slaughterhouse and detail the visceral laparotomy, carcass preprocessing, and deboning robot technology. In this study, we examine slaughterhouse automation equipment and technologies, focusing on optimizing the processing lines, the direction of application, and the hygiene of robot technique. We hope this review will provide insight into slaughterhouse automation for decision making in the slaughter industry.

## 1. Introduction

The quality of meat is greatly impacted by the handling procedures before [1,2] and during the initial step of the muscle-to-meat process, which is slaughter [1,2,3,4]. Optimization of the slaughter process leads to substantial economic benefits for the slaughterhouse by improving product quality and reducing waste. According to the Danish Meat Research Institute (DMRI), which surveyed livestock packing centers in Korea, about KRW 7.6 billion can be saved per center per year via process optimization during slaughter [5]. According to UN world population prospects statistics, the world population is expected to increase from 8 billion in 2023 to 9.8 billion in 2050 [6]. As the population increases, it is expected that the structure of the livestock farming industry will naturally change from small-scale to corporate or full-time farming. Accordingly, domestic slaughterhouses are being modernized and scaled up to meet increasing demands and improve hygiene standards. In Denmark, for example, 69 domestic slaughterhouses operated in the 1970s, but this has been concentrated into 13 large-scale operations since 2015. The Korean slaughter industry shows a similar pattern, with restructuring due to competition that is expected to accelerate further if a national base slaughterhouse is designated [5,7]. As of 2020, Korea boasts 81 slaughterhouses for beef and 86 for pork [8]. The slaughter volume of pork in 2014 was 15.68 million animals, an amount that can be processed by 17 large-scale slaughterhouses [5]. It is predicted that, by 2035, the number of pork slaughterhouses will be reduced from 86 to 40, as 15 large packers, 15 regionally specialized slaughterhouses, and 10 general slaughterhouses would be adequate [5].

The work environment of slaughterhouses are cold, damp, and noisy, which is harsh for workers [9]. In addition, slaughterhouse labor involves the usage of knives and is associated with about three times the frequency of industrial accidents seen in other industries [10]. In the United States, meat-packing industry workers exhibit injury and disease rates 2.4 and 17 times, respectively, those of the general working population [5,11]. Moreover, workers must be well-trained and highly skilled because work efficiency and meat quality vary depending on the skill level and condition of each worker [12]. As a result, the rate of new workers entering slaughterhouses is expected to decrease, worsening the labor shortage.

To overcome labor issues, automation processes have been introduced to large-scale slaughterhouses worldwide, and complete automation is in progress. Automated slaughterhouses are already in operation for large companies such as Danish Crown and Tyson [11,13]. Automated slaughterhouses use advanced technologies involving robots, non-destructive sensing, data transmission, and real-time process monitoring. Some European and American companies have introduced grading devices and quality measurement processing machines for beef, pork, and sheep carcasses. In addition, the boning of beef, pork, sheep, and chicken carcasses with a robotic arm has become feasible [14]. In Europe and Australia, the rate of slaughterhouse automation is high due to high labor costs [15]. Danish Crown has gradually developed a highly automated slaughter line at its Horsens, Denmark facility since 1988. Marel’s F- and M-lines, based in Iceland, have been installed in more than 350 pork slaughterhouses worldwide and are based on complete or partial lines with capacities of up to 1400 carcasses per hour [11,16]. In the past, large investment costs were required to introduce automation processes, and many companies were reluctant due to cost reasons. However, the labor-intensive slaughter and meat distribution industries were hit hard by the COVID-19 outbreak in 2020, as a high density of workers in a cold and humid environment led to disease outbreaks around the globe [17,18,19,20,21,22]. Hobbs (2021) predicted that automation and digitization would reduce damage during this pandemic; accordingly, the installation of automated devices and robots has increased since the pandemic began.

In slaughterhouses that process red meat, carcasses are large, and equipment must be able to cope with the complexity and size of the work. Moreover, the characteristics of meat vary with species, breed, rearing conditions, feed diversity, carcass-splitting method, and occurrences of abnormal anatomy. As such, low-cost sensors, software, and algorithms must be developed to guide robots. Each slaughterhouse is unique in structure and scale, and conditions such as lighting and humidity can vary. Therefore, it is difficult to apply a single system to all slaughterhouses. In addition, equipment and procedures must accommodate the unique set of grading and quality standards considered important by the country. To address these limitations, this study was designed to investigate the current slaughterhouse automation robot technology. Based on our findings, we discuss important considerations for the modernization and automatization of slaughterhouses. We have aimed to review the technologies required for each process, highlighting currently commercialized products. This study also introduces hygiene issues to be considered when introducing robots in slaughterhouses. This study was intended to provide an updated description of robot technology for slaughterhouse automation. It is hoped that this study will be helpful to managers and decision-makers at slaughter sites when introducing robot technology and various sensing technologies.

## 2. The Robotic Slaughterhouse Processing Line

**Figure 1:**
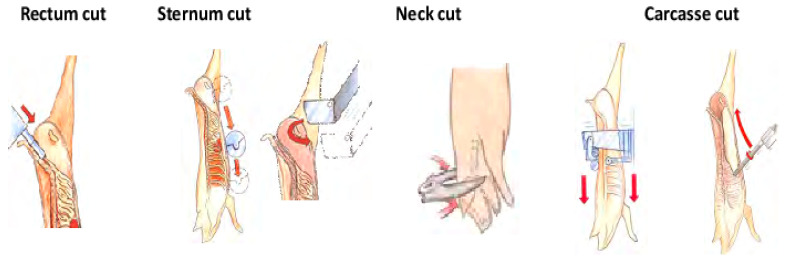
A robotic auto-processing technique for pork carcasses [24] (From Matthieu et al. Robotic solutions for meat cutting and handling. Reprinted with permission of Matthieu et al. Copyright © 2014).

The slaughter process differs slightly by species and country but is generally divided into stages of stunning, bleeding, skinning, gutting (evisceration), and carcass cutting. Errors in any one step of the slaughter process affects subsequent steps [23]. The slaughter processes for pigs and cows differ slightly due to the characteristics of the animals, but transportation, mooring, stunning, bleeding, skinning or hair removal, gutting, splitting, washing, and grading are common. In this section, we examine robots and automation technologies used in slaughter lines (Figure 1).

### 2.1. The Abdomen and Brisket-Cutting System

Evisceration, the removal of organs from the body cavity of a slaughtered animal, can lead to carcass contamination. This is due to the delicate nature of the visceral membrane of the carcass. Recently, there have been advancements in the automation of evisceration process, particularly for poultry [25]. However, the evisceration process for pork and beef is still not fully automated [11]. Partially automated robots that perform rectum removal, H-bone incision, leaf fat removal, and carcass splitting have been developed [26,27]. To eviscerate the intestines of the digestive system, commonly known as white viscera, the rectum must be removed as a pretreatment process.

#### 2.1.1. Pork Carcasses Abdomen-Cutting System

**Figure 2:**
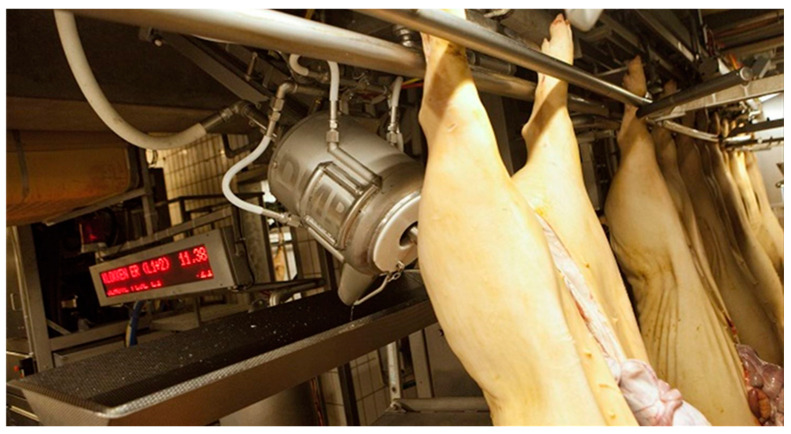
The DMRI bung dropper [34] (Reprinted, with permission from DMRI, Danish Technological Institute).

Industrial robots have been introduced to slaughterhouses as a pretreatment tool for evisceration [28,29]. To date, companies that have commercialized automatic carcass-cutting technology include Marel (Gardabaer, Iceland), Frontmatec (Kolding, Denmark), Scott (Dunedin, New Zealand), and the Danish Meat Research Institute (Taastrup, Denmark) (Figure 2). Commercially available systems can process carcasses at speeds ranging from 350 to 650 heads per hour. Systems for gutting have also been developed, mainly focusing on pork and sheep carcasses. In the past, studies on rectum removal, aitchbone cutting, and brisket-cutting techniques were conducted separately [30,31], but recently, a system that integrates these steps into one process has been developed and sold.

Robot systems for evisceration comprise a measurement station, a processing station, and a control unit [32]. In the built-in processing line, the carcass must be recognized and tracked, and the incision path through the sensor must be carefully controlled. Next, the end-effector, such as the saw blade of the processing unit, moves accurately to the starting point and proceeds with incision. In the case of beef, pork, and sheep carcasses, the trajectory and range of movement of the robot must be wide when removing the intestines [28]. In the case of internal organ removal, robots operating within a specific range, e.g., with a low degree of freedom (DOF), have been used. However, as prices of the robot arms decrease and accuracy increases, robots with a high degree of joint freedom, such as 6-axis (6-DOF) or 7-DOF arms, are gradually being introduced into the slaughter process.

Recently DMRI developed bung droppers for pork and sheep carcasses [33]. For pork carcasses, the bung is grabbed by a vacuum cup and pushed into the mesentery, in which it is wrapped and secured. It was reported that the bung dropper effectively reduces 50% of contamination in pork slaughtering process compared with manual operator handling [34] which can operate 900 carcasses per hour. This equipment keeps spreading to Denmark slaughterhouse currently. Due to the anatomical difference between lamb and pork, the equipment had to be optimized to be used for sheep carcasses. Now the equipment is using in fully automated Australian lamb slaughter lines. Frontmatec and Marel currently commercialize rectum removal robots for pork carcasses, but such devices have not been disclosed in academic reports. However, according to a company report, the detection of pork carcasses for rectal removal utilizes a 3D camera or laser sensor and proceeds with sex classification to determine the proper method of rectal removal. The robot then pulls out the rectum by applying a vacuum attached to the robot arm. It has been reported that the processing speed of these commercialized robots to date is 550–650 carcasses per hour, and that pork carcasses in the range of 60–140 kg can be processed. After each carcass treatment, cross-contamination is prevented through immediate disinfection of the robot arm. To date, no researched and commercialized robotic system exists for automating the removal of guts from beef carcasses. Therefore, studies on the robotization of the beef carcass, besides pork and lamb carcass robotization, are also continuously needed [24].

#### 2.1.2. Carcasses Brisket-Cutting System

Brisket-cutting technology is required to remove the respiratory system and heart, which are considered red viscera. Brisket cutting is generally performed after abdomen cutting. In brisket cutting, the intestines must not flow down and drag on the ground, the end-effector must not penetrate the diaphragm, and the production of bone dust must be prevented [30,31]. Condie et al. (2007) used a 2D laser scanner and video to configure measurement during the brisket cutting of sheep carcasses. Furthermore, Singh et al. (2012) used a laser sensor to measure the distance to the neck during sheep carcass brisket cutting. For measuring the horizontal distance to the brisket, they used an ultrasonic sensor. A study on a small carcass brisket opener robot reported a system in which an end-effector with a blade cuts in a straight line along the sternum just below the diaphragm before exiting through the neck [35]. In this study, the brisket opening test was conducted in a low-speed situation where the beef carcass was fixed and in a high-speed situation. The authors mentioned that it did not achieve the production success target rate (99%), since in that pilot slaughterhouse, the backs of a large number of carcasses were broken during hide removal, which causes the carcasses to become twisted. Thus, the system cannot straighten the carcass and complete the cut in every instance. The research team mentioned methods that can overcome this challenge, such as using a higher degree of freedom (DOF) and high-power robots. Increasing DOF in a system refers to adding more variables or control inputs that determine its behavior [36]. However, it has both strengths and weaknesses. Advantages of increasing DOF include increased versatility, improved precision, and enhanced functionality. On the other hand, the drawbacks include increased complexity, decreased reliability, and higher cost. Hence, the decision for the DOFs increment need to be considered with the trade-off between pros and cons [37].

### 2.2. The Head and Jowl Cutting System

Robot technology for carcass preprocessing procedures, such as the removal of the head area from pork carcasses, leaf lard removal, carcass splitting, and jowl cleaning, has been commercialized. To remove pig heads, the Marel M-line neck cutter (MNC, Marel, Gardabaer, Iceland) and the AiRA RNC Neck Clipper are used and can process up to 650–750 pork carcasses per hour. These products can cut off the head just above the ears of the carcass [11] and use 3D image system technology for object recognition in this process. Pork carcasses leaf lard discarding systems include the AiRA RLR Leaf Lard Remains Remover and a similar Marel product (M-Line leaf lard remover, Marel, Gardabaer, Iceland). These robots can handle 650–700 heads per hour and use a removing roller to remove lard residue and leaf lard from left and right pork carcasses at the same time. Moreover, robotic products for jowl cleaning and hock cutting of pork, beef, and sheep carcasses can process 450 heads per hour (Jowl Cleaner APT4, Frontmatec, Kolding, Denmark) and can produce more uniform toe clipping than that accomplished manually. Hock-cutting systems utilize a 3D vision system to identify the hock and detect the individual height of each carcass [38]. In addition, 6-DOF robot products have been reported to be feasible for the hock cutting of beef (450 heads per hour; JR-50, Jarvis, Middletown, CT, USA) and sheep (600 carcasses per hour; AHC-2, Jarvis) carcasses.

### 2.3. Intestine Control

**Figure 3:**
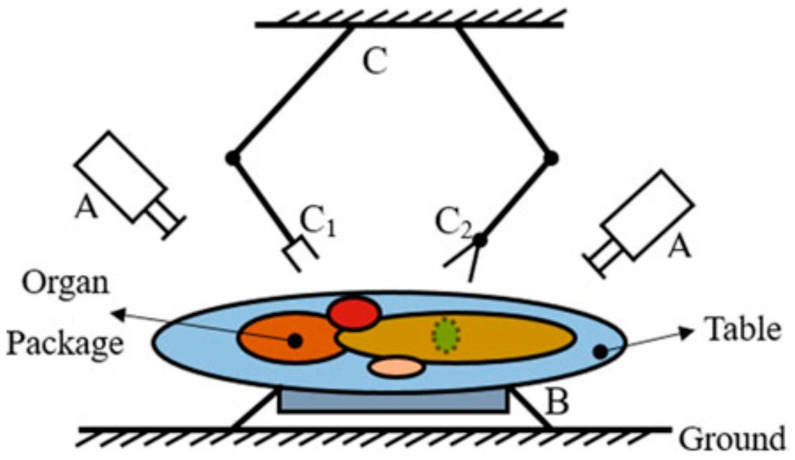
A robotic system for ovine offal harvesting. (A) Machine vision (camera); (B) a sorting device; (C) a dual-arm robot; (C1) a vacuum gripper; (C2) a specific cutting device [40] (From Zhang et al. Discussion of Soft Tissue Manipulation for the Harvesting of Ovine Offal. Reprinted with permission of Springer Nature, London, Copyright © 2021).

In general, the evisceration process is not well automated and relies on manual work. Recently, however, as human labor has begun to become insufficient, studies are being conducted to automate evisceration and quality evaluation [39]. To date, there is no commercialized system for eviscerating beef and pork carcasses. Still, Zhang et al. (2020) proposed that evisceration of sheep carcasses could be achieved through machine vision, sorting devices, and dual-arm robots (Figure 3) [40]. First, the type and location of each internal organ must be identified through a machine vision system using a camera. Two end-effectors then separate the intestines, one with a vacuum gripper with a small attached suction cup and the other with a cutting device. The evisceration system operating process first lifts the identified single organ with a vacuum gripper and subsequently cuts the connective tissue of the single viscera with a cutting device before returning the separated organ to its designated position using the vacuum gripper. Appropriate soft robot technology [41] and grippers that can reduce the impact on the intestines will be needed to separate the intestines automatically. In addition, to fully automate the system, an evaluation method must be added to analyze whether the evisceration proceeds without problems.

### 2.4. The Auto-Splitting System

The splitting process for pork and beef carcasses enhances the convenience of handling and cooling speed [35]. Exposing the spinal vertebrae and cord by the splitting process helps to inspect the potential presence of abscesses within the carcasses, which is required by food safety legislation. Automatic splitting system become popular in modernized large-scale slaughterhouses while manual splitting saws still have been used in small-scale slaughterhouses. The accuracy of splitting is critical because incorrect segmentation can cause problems in later carcass inspections or subsequent automated processes [42]. Previously, the division of beef or pork carcasses was performed by a person using a rotary saw blade operated on a hydraulic platform, but this can lead to uneven splitting. However, automation using a robot arm has recently been in progress, increasing uniformity [43]. Li et al. (2003) demonstrated carcass splitting using an ABB IRB 6600 robot (ABB, Zürich, Switzerland) [44]. The detection of the spine was performed using ultrasound images to generate transverse coordinates and control the horizontal position and rotation angle of the saw blade. In this type of system, ultrasonic feedback data automatically correct the saw position and rotation about its axis while the robot arm moves along the spine [45]. The Australian Meat Processor Corporation Limited (AMPC, 2017) mentioned the need for appropriate sensing technology to enable accurate segmentation when constructing a bipartite carcass system [43]. The report described the need to segment all carcass types properly and process at least 135 beef carcasses per hour. Fully automated splitting systems currently exist for beef and pork carcasses and are produced by companies such as Frontmatec, Jarvis, and Marel. These products are known to process 450–900 heads per hour in the case of pork carcasses and 195 carcasses per hour in the case of split beef carcasses. However, field users have reported cutting deviations from the exact center of the carcass, indicating a need to improve the accuracy of cutting while minimizing the generation of bone dust [42,46].

### 2.5. The Automated Primal Cutting and Deboning System

In the case of boning robots, progress has been made in the line that handles small-sized carcasses, and the technology has been commercialized in the order of sheep, pork, and beef carcasses. In this study, equipment for primal cut and trimming-related equipment and technology utilization status is reviewed.

#### 2.5.1. Primal Cutting

Scott Automation, which is famous for its automation process for sheep carcasses, developed a system that maps the bone position of the carcass via X-ray and laser-based 3D measurement of the sheep carcass. The X-ray information is transmitted to the boning room module, and the exact cutting position and angle are calculated. The entire system is divided into six systems: X-ray grading, X-ray primal system cuts, the forequarter system, the middle system, the hindquarter system, and the knuckle tipper system. After all steps, the sheep carcass is completely processed into meat parts. This system processes 600 carcasses per hour [11]. Frontmatec has also commercialized a system capable of dividing 300–400 heads per hour. Details of the technique can be found in reviews written by Joshi et al. (2017) and de Medeiros Esper et al. (2021) [11,45].

Commercialized products have been developed by Frontmatec, E + V Technology, and Marel for pork carcass splitting. Frontmatec produces a cutting line-type system (AGOL-800) that divides half pork carcasses into three parts (leg, middle, and fore-end). The system is capable of processing up to 800 heads per hour and aligns the carcass using a vacuum dropper for accurate cutting. The E + V Technology pork carcass automatic primal cutting system uses a robot arm and an automated conveyor belt-like cutting system. The E + V Technology VRCS 2000 utilizes a robotic arm to perform the primal cutting of pork carcasses and features a rotary motor before the robot cell so that the carcass can always be put into the robot cell in the same direction. Both Marel and E + V Technology also produce conveyor belt-like cutting machines that use two saw blades to cut and divide carcasses into ham, belly, and shoulder sections. The characteristics of the carcass are measured using a camera, and the position to be cut is visualized via laser. The Marel system (Primal cutter, Marel, Gardabaer, Iceland) is said to be able to process 600 carcasses per hour.

Beef carcasses are difficult to handle due to their large size (200–1000 kg) and variations in characteristics based on in breed, age, and type (bull, steer, heifer, cow, etc.). Therefore, dividing beef carcasses into cuts has been a difficult process to automate [32]. Only a few systems exist for the large-section cutting of beef carcasses, including robotic technologies for carcass processing. In a patented system developed by Texas Beef in 1993, a cutting path is created using data from X-ray images, 3D image sensors, and ultrasonic sensors for refrigerated beef carcasses [47]. The robot then proceeds with a large division using high-pressure water jets and an abrasive agent to cut the meat and bone, respectively, and air jets to push the meat from the cut area. Although the patent has been filed, it is unknown whether the technology has been incorporated into current systems [29,40].

Scribing is the initial operation of cutting the side of the carcass. This operation results in high potential costs regarding labor use and availability due to low accuracy and serious safety risks [48]. According to the Australia meat processor corporation (AMPC), the initial scribing research was performed by inputting the cutting path on the touch screen semi-automatic and cutting with the robot arm [49]. However, it was reported that this method was abandoned because it did not meet the technical capabilities of the touch screen and other vision systems at the time. Recently, it has been said that the technology is being reconsidered by AMPC according to the development of touch screens and vision systems [49]. In an early scribing automation study, Li and Hinsch (2003) developed a sensing technology that determines the proper cutting position on a beef carcass [48]. They used a 3D imaging camera and carcass identification system placed at a distance of 3 m from the carcass to determine the location and depth of the ribs and detect the contour, thoracic cavity, and side of the carcass. However, this research does not appear to have been commercialized as a product. Recently, Scott developed a commercial scribing robot system based on a robotic arm. This system utilizes dual-energy X-ray absorptiometry (DEXA) technology, a 3D scanner, and a color camera to determine the bone position and cutting path. The system has been reported to process 240 carcasses per hour and reduce labor requirements by 2–3 workers per shift.

**Figure 4:**
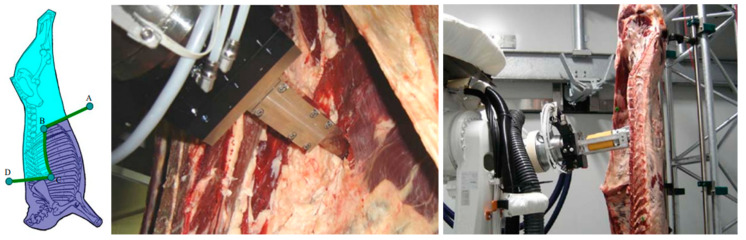
Beef carcass industrial processing using robots. The Z-cutting and prototype cutting system [51] (From Guire et al. Robotic cell for beef carcass primal cutting and pork ham boning in the meat industry. Reprinted with permission of Emerald Publishing Limited, Bingley, Copyright © 2010).

The Z-cut is a kind of primal cutting method on beef carcasses. This cutting method first cuts at the 13th rib, between the 5th and 13th rib, and through the vertebra column at the 5th rib [50]. Guire et al. (2010) demonstrated the Z-cut for left and right beef carcass division into quarter parts using a robot arm in SRDViand (Systemes Robotis’es de D’ecoupe de Viande) research [51] (Figure 4). For Z-cut automation, the research team first obtained image information of carcasses using structured light and then determined the cutting path based on these data. The cutting path was modified based on a set of variables connected to the knife, along with the position and orientation of the bone in space and contact force. They reported that adjusting the direction of the knife greatly influenced the results. Moreover, the use of a 6-DOF robot arm with integrated visual and force-type external sensors enabled accurate Z-cut execution.

#### 2.5.2. Meat Deboning and Trimming

**Figure 5:**
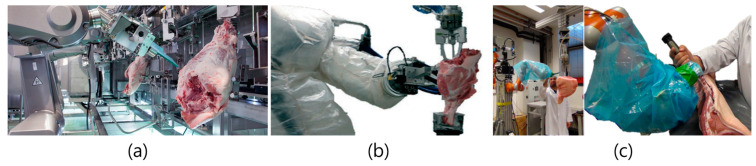
A pork meat deboning robot technique. (**a**) Mayekawa Hamadas RX (Reprinted with permission from Mayekawa, Japan); (**b**) a ham deboning using a robot [52] (from Subrin et al. Analysis of the human arm gesture for optimizing cutting process in ham deboning with a redundant robotic cell. Reprinted with permission of Emerald Publishing Limited, Bingley, Copyright © 2014); (**c**) a meat deboning using co-robot [55].

Carcass deboning is one of the slaughterhouse processes that most benefits from automation because the cold working conditions can predispose workers to musculoskeletal disorders. Professional workers must manually identify the characteristics of the carcass and the location of the bones through visual and tactile indications. Robots must therefore obtain separate signals for sight and touch to achieve the same performance. Robots with more than six axes of freedom are required for boning automation, and such systems are being introduced in Japan, New Zealand, and Denmark [52]. A representative commercial system is the Mayekawa HAMDAS-RX (Tokyo, Japan) bone equipment technology (Figure 5). The system removes the hip and tailbones and can process 500 hams per hour. A similar system, the WANDAS-RX, is capable of front-leg boning and can process 600 units per hour. The HAMADAS-RX boasts a high boning rate with an average meat loss as low as 60 g per one ham part [53]. The system uses X-ray-based sensors to measure the location of the bones in the hind legs, and even includes functions for distinguishing between the right and left legs and measuring total bone length to account for the difference in length between the entire calf bone and thigh bone. A research team examining the automation process of a ham boning robot based on analysis of human arm motion conducted an in-depth study of skilled workers to develop 7-DOF robot arms [52,54]. Subrin et al. (2014) placed a force sensor between the robot and the end-effector to detect the interface between meat and bone when constructing a robot system for hindlimb boning [52]. Path control [29] and force control are essential elements of a slaughter robot. The theoretical trajectory of the robot is first programmed and then repositioned through force control, allowing the robot to naturally recognize its position and respond spontaneously to the force it feels [54]. The repulsive forces of muscle, fat, and bone are different, and this must be reflected in robot operation. Kinematic redundancy management and additional criteria in solution selection are necessary to ensure the mobility of pathways [54]. The threshold and weight criteria obtained through the human arm model were used in the path optimization plan of the robot arm. However, this is not yet used in any commercialized product.

Recently, research has been conducted to increase the convenience of manual boning by using collaborative robots. Physical human–robot interaction (pHRI) describes the use of wearable robotic systems that combine human cognition with robotic accuracy. Maithani et al. (2021) demonstrated detailed pork cutting using a collaborative robot [55]. Prediction and force amplification strategies were used to understand the intended direction of blade movement. According to the results of this study, the collaborative use of human and robot labor reduced manual force requirements by about 30%.

As the development of automation technology for carcass boning progresses, sensors are attached to working knives to relay information to robots. Mason et al. (2022a) defined the criteria for smart knives and introduced associated technologies. Optical sensing, near-infrared spectroscopy, electrical impedance spectroscopy, force sensing, and electromagnetic wave-based sensing can be applied to smart knife technology [56]. In another study by Mason et al. (2022b), a smart knife was installed on a robot arm to demonstrate the feasibility of a smart knife based on sensor feedback for use in a meat factory cell [57]. The smart knife installed on the robot showed an average error of 1.78% for contact detection and one of 7.66 ± 1.45 mm for depth detection.

**Figure 6:**
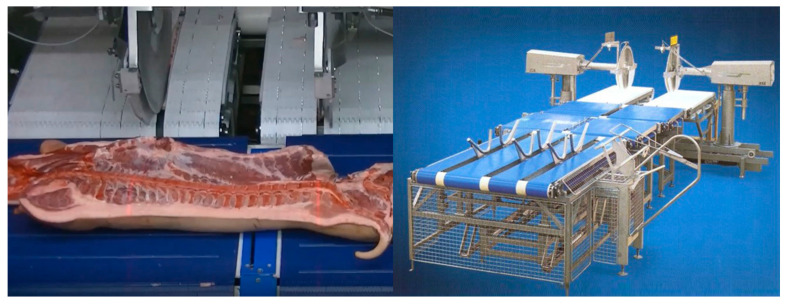
The E + V Technology VCCS 2000 system for the automatic primal cutting of pork carcasses (Reprinted, with permission from Hinz A of E + V technology, Copyright © 2023).

In addition to pork hind leg boning, the automation of line-based pork boning processes is currently feasible. Frontmatec produces various pork automatic boning machines, including a system capable of processing up to 1000 pork middle parts per hour (Figure 6). In this system (AMBL-1000), the middle part of the pork carcass moves along the conveyor and is automatically separated into loin, belly, and backbone sections by a rotating saw blade. Another machine trims sirloins and can automatically process back fat and skin from 1000 sirloins per hour. An automatic system has also been developed for pork belly boning and utilizes a 60,000 psi water jet to cut through the meat. The length, width, thickness, and weight of the pork belly are measured to determine the cutting path. Afterward, two 6-DOF robots use water jets to shape the pork belly. The system can process 1400 pieces of pork belly per hour. In addition, the automatic rib puller can separate 1500 ribs per hour, using 3D-based measurement to determine its cutting path and removing the bones with a 6-DOF robot arm. While the ribs are being removed, the part being processed is fixed in place on the conveyor belt using a vacuum.

**Figure 7:**
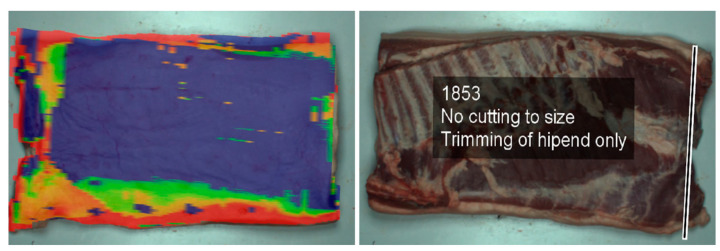
An AR-assisted technique for base trimming [59].

Recently, visualization systems based on augmented reality (AR) have been introduced to increasing worker efficiency (Figure 7); this has been demonstrated with pork belly fat trimming [58]. The AR-based system relies on the Creator software suite and the Junaio display channel to evaluate the potential yield of pork belly and account for biological heterogeneity, optimizing the manual trimming process and improving yield. The workflow first proceeds with a computed tomography scan of the pork belly, which then directs the creation of a corresponding 3D mapping image. Surface information in the 3D images is mapped and traced into 2D images [58,59]. The Microsoft HoloLens product can then be used to deliver this information to the operator [60]. Currently, the system has the advantage of being able to measure product information and provide visualization data to workers. However, AR devices can cause workers to feel disoriented or dizzy after extended use [61]. Therefore, important considerations are required when discussing technology development and utilization in this field.

Associated with beef fat trimming, one R&D project has focused on using ultrasonic sensor measurements to accurately separate lean meat from fat by guiding a tool through a mathematically based path generation process within a robot program, using the trajectory planning features of the embedded software [62]. The dimensional information provided by the ultrasonic sensors helps locate the interface between meat and fat, allowing the specified thickness of fat to be left on the lean meat [27]. The technology has potential for development and commercialization, but it is judged that there is no available information related to it later.

### 2.6. The Meat Factory Cell (MFC)

Modern line-type slaughter processes boast high productivity but have substantial investment costs, low process flexibility, and relatively low food safety confidence [63]. These issues are also linked to food security problems, and modern technologies are needed to efficiently utilize essential food resources in the surrounding area [63]. Mason et al. (2021a) suggested that full automation is feasible only in facilities processing more than 600 heads per hour (about 25,000 heads per week) [64]. Most existing small slaughterhouses are yet to meet the demand to warrant full automation.

**Figure 8:**
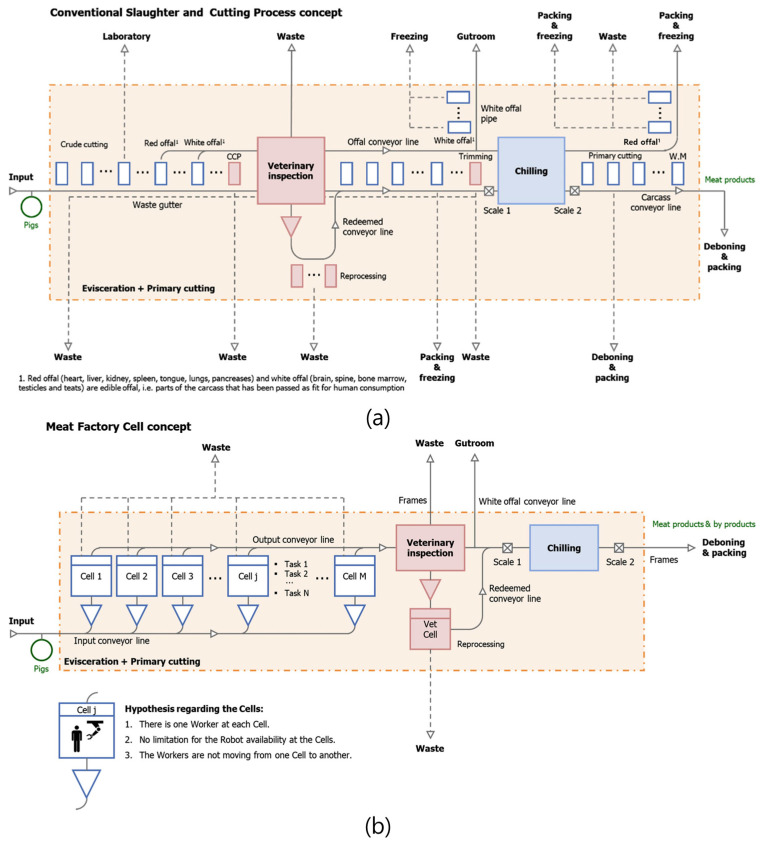
(**a**) A line style conventional meat processing line; (**b**) the meat factory cell processing concept [65,69] (from Valente et al. Life cycle sustainability assessment of a novel slaughter concept. Reprinted with permission of Elsevier, Amsterdam, Copyright © 2020).

**Figure 9:**
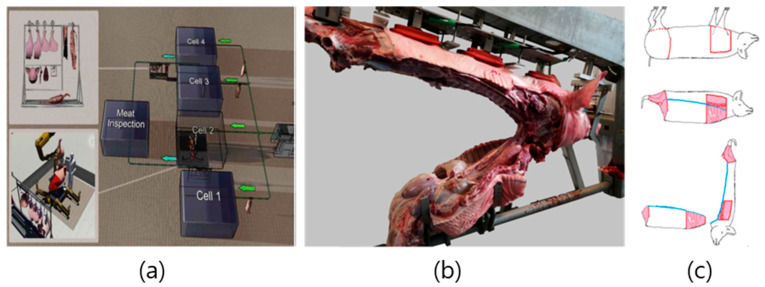
(**a**) The meat factory cell concept [69] (From Valente et al. Life cycle sustainability assessment of a novel slaughter concept. Reprinted with permission of Elsevier, Amsterdam, Copyright © 2020). (**b**) A carcass handling unit: The limbs have been removed, and the belly and ribs sawed approximately 12–15 cm from the spine. The truncus has also been lifted. The trachea, esophagus, and some soft tissue are available for the butcher [67]. (**c**) A schematic of the meat factory cell cutting pattern [67].

Existing slaughterhouses proceed from the slaughter to the disassembly of carcasses along the slaughter line. However, an MFC refers to a parallel, independent cell process rather than a conventional line-type process (Figure 8 and Figure 9). MFCs show automation potential for small-scale factories that meet food hygiene regulations [63]. Siles (2018) compared ordinary slaughterhouses with MFC-concept slaughterhouses in their research (Figure 8a,b) [65]. MFC technology has recently been implemented for primal cut production from pork carcasses [63,66,67]. Unlike individual processing slaughter lines, MFCs allow for simultaneous evisceration and large-section meat cutting. Recently the ‘RobutcherEU’ project proved the concept of MFC by demonstrating auto-removal techniques for both forelimbs and hams without any manual operation [68].

MFCs exhibit three major differences from general line-type slaughterhouses. First, work is performed in cells (one boning room), not in lines (Figure 8b and Figure 9a). Second, the slaughtering and carcass-splitting processes are combined. Finally, primal cut cutting occurs externally, without removing internal organs first [67]. MFCs, therefore, exhibit reduced contamination compared to general line processing systems because internal parts of the carcass are not touched. The MFC system can significantly reduce exposure to fecal contamination from intestinal contents by removing the target sections—forelimbs, hindlimbs, the neck, and loins—instead of the digestive tract [63]. The MFC process is currently in the research stage, and fully or semi-automatic MFC methods are being studied to increase the applicability of this process [64,67]. In the case of semi-automation, humans and robots cooperate in such a way that the robot performs heavy or repetitive work and the human worker performs cutting work. Mason et al. (2021a) describes a case in which two ABB IRB4600 series robots are used in an MFC for full automation with payloads of 40 and 60 kg at working distances of 2.55 and 2.05 m, respectively [64]. One of the two robots uses a gripper to grab the front or hind legs, and the other uses a knife to divide the carcass. The system also utilizes a 3-DOF carcass handling unit (CHU) to simplify the cutting process using vacuum grippers arranged along the back of the carcass and mechanical clampers on the head of the carcass. The system helps stretch the trunk of the carcass during rib cutting, effectively helping the viscera to separate from the carcass by gravity. Vacuum grippers can hold fresh carcasses for 20–30 min [64]. A recent MFC-type automation research project is in progress and has published some useful data regarding the automation process. For example, an open dataset for robotic MFC boning provides step-by-step image data for the removal of the shoulder, ham, and rib sections of 25 pigs. The dataset provides information regarding red, green, blue, and depth (RBG-D), intrinsic, and extrinsic parameters [70]. Further studies have used these data to specify the orientation and gripping points of the porcine carcass [71,72]. In this study [72], RGB-D data taken from six different directions and a U-net-based deep learning model were utilized. Although the accuracy of the grip point on the leg was slightly different between the Danish and Norwegian cutting styles, mean average precision (mAP) values were reported in the range of 0.9504–0.9831, and the distance error was within 1.5 mm. de Medeiros Esper et al. (2022b) studied the RGB-D image calibration method to estimate the position of an object in the CHU and reported high performance using the tool center point for calibration [71].

Sødring et al. (2022) experimented with the quality evaluation of pork carcass processing using the MFC method [73]. They determined that quality and sensory characteristics were comparable to those of products from the traditional line method. Moreover, the application of proper packaging and cooling could yield MFC products of similar or, in some cases, superior quality to that of existing products [73]. Alvseike et al. (2018) reported that fecal contamination due to intestinal content is improved in the MFC methods when compared to traditional systems because the intestines of pork carcasses are removed first [63]. In general, in the slaughtering process of pork, flame-throwing is performed to burn the hair on the surface of the carcass. However, they found it difficult to completely remove feces exposure on the surface of pork carcasses through burning and polishing via flame-throwing. For this reason, Mason et al. (2021b) reported the need for additional sensing research on the external contamination of carcasses [74]. Until the MFC method is commercialized, the continuous development of food regulatory plans and systems is required. However, the MFC system is considered feasible for the automation and sustainable development of small-scale slaughterhouses with low processing volumes [64,74]. Valente et al. (2020) conducted a life cycle sustainability assessment of MFCs. As a result, MFCs showed no significant difference from existing slaughterhouses regarding environmental impact [69]. MFCs were more economical than the existing slaughter method, suggesting it is a viable alternative. In addition, it was reported that the social effects could be lower due to the lower risk of injury and accidents compared to the conventional slaughter process.

## 3. Robotic Techniques for Slaughterhouses

### 3.1. Hardware

#### 3.1.1. Robotic Grippersand End-Effector Designs

**Figure 10:**
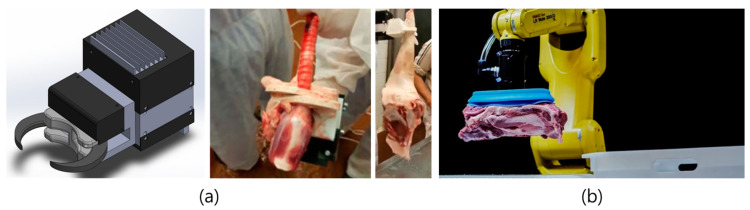
Different types of automated grippers for meat processing. (**a**) a dual propose gripper [75] (from Takács B. Inner organ manipulation during automated pig slaughtering-smart gripping approaches. Reprinted with permission of IEEE, New York, Copyright © 2021). (**b**) A DMRI vacuum-type gripper for the meat processing line (Reprinted with permission of DMRI, Danish Technological Institute).

As slaughterhouses become automated, grippers and end-effectors are key technologies to be considered (Figure 10). Slaughterhouses currently use tong-like [75,76] and vacuum grippers [64,77]. Tong-type grippers, in particular, tend to be used extensively in the industry [11]. To facilitate storage and transportation in the meat processing line, several loins or hams are tied together to make a lot called a “Christmas tree,” so-named for its resemblance to its namesake. Wu et al. (2016) used a pneumatic gripper to suspend a pork loin in the form of a Christmas tree lot [76]. This gripper featured two jaws and was lightweight and easy to mount on the robot end-effector. Takács et al. (2021) developed a multi-purpose gripper for the evisceration and manipulation of the external limbs of large animals [75]. The system is designed to detect force, torque, and slip and to observe any slipping of the target sample by installing a single-focal length camera on the gripper. This research is a research document developing a gripper that can be used in robots at MFCs and is designed to grip soft tissues easily.

Jørgensen et al. (2019) developed a method for fixing meat using a vacuum gripper. In this study, a rolling lift method was used to allow air to flow under the meat pieces and prevent sticking to the vacuum gripper [77]. Ross et al. (2022) investigated technologies to improve slaughterhouse grippers and concluded that simple vacuum grippers were suitable for slaughter lines [78]. Danish Crown also uses a vacuum gripper robot system developed with DMRI to transport heavy meat from slaughterhouses [79]. The suction cup in this system exhibited no evidence of bacterial propagation during 8 h of working time. A detailed description of a robotic gripper for slaughterhouses is provided by Ross et al. (2022) [78].

#### 3.1.2. Hygiene and Sanitation

Robotic end-effectors used in slaughterhouses may include knives, rotary saw blades, vacuum droppers, or probe-type sensors for pH measurement, all of which pose a risk for cross-contamination. Smid et al. (2012) tracked *Salmonella* contamination in a Dutch pork slaughterhouse equipped with splitter and belly opener robots. They found that *Salmonella* bacteria found within the carcasses likely originated from flora on the carcass splitter [80]. This finding indicates that immediate disinfection is required to prevent cross-contamination after the processing or measurement of one carcass is completed. End-effector disinfection methods may vary by company but usually depend on hot water treatments. In one method, a hot water treatment module is designated, and when the processing of one carcass is completed, the robot arm returns to its designated module and proceeds with disinfection. In another method, two identical end-effectors are installed on the robot arm such that the dirty end-effector is disinfected with hot water while another clean tool processes another carcass. After disinfection, the now clean end-effector shifts back to process a carcass while the newly dirty end-effector moves to be disinfected. However, another method involves the exchange of a soiled end-effector for a disinfected blade for each new carcass. Used blades are disinfected sequentially while other blades are in use, and blades are continuously cycled between use and disinfection. In the case of splitter and band saw robots, the water and steam chamber continuously emit water and steam during the cutting phase. This will help reduce bone and meat residuals by cleaning the cut surface. To date, no disinfection standard exists for end-effectors, but existing food sterilization laws can likely be modified and applied to robots. According to the Korean livestock product sanitary control act, Article 29 (physical examination), “in the work line, hot water of 83 °C or higher must be installed at regular intervals to disinfect knives used for carcass dismantling work and inspection” [81], but this article can be modified as statutory rules for the sterilization of robot end-effectors. For example, these laws and regulations can be applied to the rule of the disinfection time of the end-effector, the temperature condition of hot water during disinfection, or the accessory replacement cycle of the end-effector. Nagel-Alne et al. (2022) suggest that functional requirements and objective standards should be established to identify practical applications for the slaughter industry [82]. The researchers suggested that red meat safety legislation in the U.S., Europe, and New Zealand, along with highly generalized global guidelines, may create unintentional barriers to innovation and new technologies [82]. In general, consideration should be given to actual and business site needs when applying technologies similar or equivalent to those regulated by law. Nagel-Alne et al. (2022) mentioned that functional requirements and objective criteria should be targeted when future meat safety laws are amended [82]. As such, future studies should investigate whether the influence of food sanitation legislation acts as a hurdle, impeding automation and innovation in the Asian meat production industry.

The slaughterhouse environment tends to be damp, promoting rust occurrence on end-effector parts. Moreover, because impact with the carcass occurs, the device must be durable enough to prevent breakage. For this reason, robots used in slaughterhouses, food factories, and semi-conductor processes must be designed according to sanitary design principles and comply with requirements defined by International Organization for Standardization (ISO) 14159 and European Hygienic Engineering and Design Group (EHEDG) document no. 8 [83,84]. Panda et al. (2023) described three rules for robot and end-effector hygienic design: (1) toxic substances, (2) microbiological contamination, and (3) discoloring [85]. (1) Toxic substances: Trace amounts of toxic substances can adhere to food and transfer to consumers during food handling by robots. Müller et al. (2014) also mentioned that, in the case of robots used in slaughterhouses or fish processing companies, certain common coatings for robot parts are avoided, and manufacturers are urged to develop materials that can make direct contact with products [86]. Therefore, all gripping devices must be made of non-toxic materials [85]. For this reason, robots and end-effectors that directly interact with carcasses must comprise food-grade stainless steel, e.g., SUS 304, or plastic materials. Stainless steel is the most suitable construction material for the hygienic design of robot end-effectors, as it is inert and corrosion-free [85]. (2) Microbiological contamination: Food processing, handling, and packaging facilities favor the growth of bacteria and fungi due to the availability of organic materials, high humidity, and temperature conditions. Keller et al. (2018) suggested that unnecessary appendages and crevices where microorganisms can propagate should be avoided in the sanitary design of robot systems, and specially designed screw heads should be used on exposed surfaces [87]. They also stated that all surfaces should be designed for easy cleaning. The gripper design should be such that there should be a minimum accumulation of food traces on the contact surfaces after each processing cycle [85]. (3) Discoloring: Sometimes, localized pressure from the gripping action of the gripper can cause food to change color due to slight changes in material structure. Minor discoloration does not affect the product’s nutritional value but may be regarded by consumers as a visual quality defect [85]. In the case of meat, discoloring effects become less than in fruit and other food products. However, grippers can sometimes damage meat products. Therefore, selecting grippers suitable for food materials and force-displacement measurements on gripper materials are also essential.

Often, a cover is placed on the robot arm to minimize potential contamination and prevent the robot from being exposed to moisture or the surrounding environment. Several companies produce covers that comply with European food regulations for slaughterhouse robots. Moreover, a company in France claims to produce robot covers (TEXPRAL B+, Advanced Systems of Protection (ASP), Nancy, France) for slaughterhouses that are resistant to high-pressure water spray, disinfectants, and abrasion. In addition to physical durability, the robot cover must comprise a food-grade protective material and be free of polyvinyl chloride. Moreover, covers used for food processing must be continuously inflated and regularly cleaned to minimize the risk of contamination and wear. To this end, Müller et al. (2014) suggested that operators maintain continuous and sufficient internal air pressure in the robot cover and remove internal condensation using air circulation [86].

#### 3.1.3. The Optimization of Sensing

Robot processing can be hindered by various causes in the meat processing environment. Slaughterhouses are damp with low temperatures, promoting the generation of water vapor, which can negatively affect robots or sensors. Furthermore, morphological heterogeneity between carcasses could trigger sensor recognition failure or end-effector path-setting errors during carcass processing. Important technologies, such as those for fixing, transporting, and guiding carcasses through the slaughter line, are required to obtain uniform data and support the carcass during processing. Sensor performance is therefore crucial to ensure the robot’s cutting accuracy, and the guard or guideline that holds the carcass steady also plays an important role in avoiding measurement or incision site errors due to accidental movement [88].

**Figure 11:**
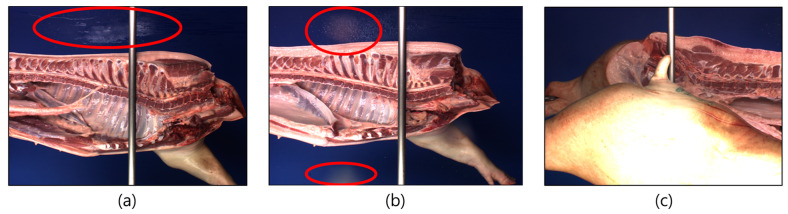
Artifact generated during image acquisition by automated pork carcass processing systems. (**a**) Image Artifact 1: blue screen spottedness (red circle location), (**b**) Image Artifact 2: an after-image (red circle location), (**c**) Image Artifact 3: an overlapped carcass image.

To perform the same processing for each carcass using a robot, when the carcass is placed into the robot work area, it must enter in the same direction, and a system that can guide it is also essential. If the carcass does not enter the same direction, it can cause problems. For example, determining a region of interest (ROI) area on the carcass becomes challenging. This naturally causes the algorithm problems, such as feature extraction failure and limits to a robot arm’s working area. To solve this problem, some companies rest pork carcasses’ backs on vertical-type conveyor systems to transport them in the same direction without rotation. Other companies use a motor to properly orient the rotation of the carcass for processing while it enters the robot cell. The E + V Technology pork carcass grading system involves hanging on the holder of a right side carcass on guard for a certain period to acquire an image of the left side carcass ROI. However, this function cannot be performed if the carcass is large enough to deviate from standard specifications or does not fit well into the programmed guidelines (Figure 11). As a result, an error occurs in the measurement, and additional data processing is needed. Therefore, designing an appropriate guide system is crucial to acquire uniform image data. Currently, the manufacturers of these systems appear to be using algorithms to restore the affected and hiding areas to compensate for misplaced carcass images.

**Figure 12:**
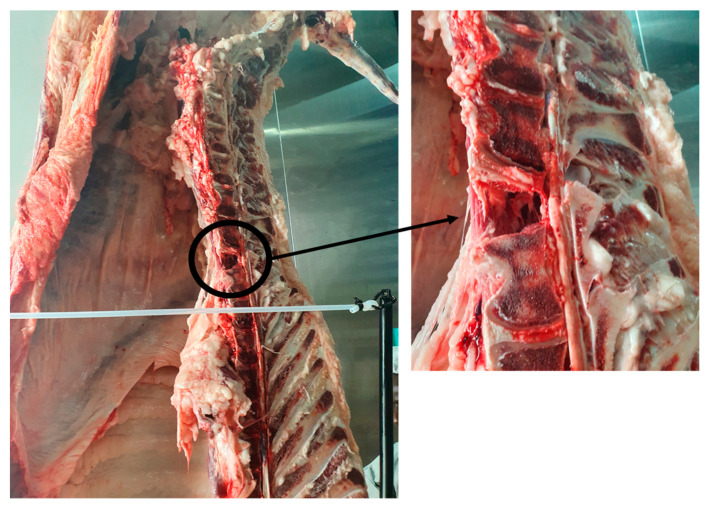
Carcass damage due to motor rotation.

In addition to transport considerations, designs for slaughterhouse robots must include technology designed to avoid damage to carcasses. Livestock carcasses are generally flexible, but the continuous impact or bending can cause damage (Figure 12). For example, the constant rotation of motors during processing can harm carcasses. Therefore, appropriate carcass control technology is also required.

### 3.2. Software

#### Robot Calibration

Robot calibration refers to establishing a transformation relationship between a coordinate system of robots, industrial cameras, and end-effectors and includes a camera, hand-eye, and coordinate system calibration of grippers or mounted tools. Ming et al. (2019) constructed an abdomen-cutting robot system for pork carcasses using a binocular vision method and a 6-DOF robot arm. Conductor recognition, image depth recognition, and position calibration were performed through binocular vision. Feature extraction based on machine learning was performed on the pork carcass image obtained after coordinate system calibration for each element. Through this, the center line of the carcass was detected. The pork carcass-cutting trajectory was then planned using the carcass center endpoint, the extracted features, and the initial point. By measuring the distance to the carcass via binocular vision, abdomen cutting was possible without damaging the viscera.

## 4. Conclusions and Future Research

Slaughterhouse automation is indispensable, considering the decreasing human resources available for labor and the increasing demand for meat products. However, a completely automated process may not be affordable for slaughterhouse managers currently due to the high initial costs and scale variations among slaughterhouses. Even when equipment is introduced, the optimization of each process takes time. For this reason, we suggest that managers need to adopt a gradual approach when optimizing and automating slaughterhouses. When introducing equipment to automate the slaughter process, economic feasibility analysis should be performed according to expected profits. Data related to economic analysis seem to be managed well on the DMRI or AMPC side. Although the economic analysis data may not be applicable to all slaughterhouses, the analysis result can be used as primary data assisting decisions for the introduction of slaughterhouse equipment. With the decrease in the population of the slaughterhouse industry, more cooperation between workers and machines is expected. In small operations, the recently developed MFC method is considered suitable for automation. However, in-depth research is required for practical application. Demand for the sanitary design of robots, along with optimized end-effector and gripper technology, is expected to increase as slaughterhouses become automated. In-depth research on hygiene for the confirmation of the cleaning level for the end-effector used in the complete automation needs to be continued. New sanitary legislation might be required to support this demand, and standards for system development need to be established and updated. EU countries and USDA agricultural marketing service, Australia AMPC, etc., have validation processes and frameworks related to slaughterhouse robot facilities. This verification process can be a reference for other countries that are newly automating slaughterhouses. This study is expected to be used for its preliminary review data when introducing automation equipment to slaughterhouses or when designating legal systems related to robots.

## Data Availability

The data are available on request from the corresponding author.
